# Obstetrics

**Published:** 1877-08

**Authors:** 


### IV. OBSTETRICS.

MENSTRUATION BY THE PEDICLE AFTER OVARIOTOMY.—W. F. Atlee. (*American Journal of the Medical Sciences*, July, 1877.) This menstruation by the pedicle has taken place several times after ovariotomy in the practice of Dr. John L. Atlee. The following extract from a letter by a lady of Morristown, N. J., upon whom he performed ovariotomy in June, 1875, will be interesting in this connection. The pedicle was very short, so short, indeed, that the clamp seemed to touch the left horn of the womb. "I shall try and write as plainly as I can just how I have been since the operation, one small place about the centre of the scar left by the clamp has never entirely healed; for some months except when discharging the exact spot was not perceptible without the closest scrutiny. Since then it has assumed the appearance of a pimple, which has increased in size, and always has a bright red color, and just before it breaks, a purplish red appearance. The discharge always occurs at the time of my monthly sickness—sometimes commencing the day previous—discharging only a few drops at a time followed by blood. The discharge is thin, clear and watery, almost sparkling—looks as though it might be sticky, but never has any appearance of corruption. I should think the discharge each time might amount to about a half teaspoonful and about double the amount of blood. Latterly it has broken at other times, then only the clear liquid; but has never failed to break at the time of my sickness since I had the operation." D. A. K. S.

Ether Spray Externally in Post-partum Hemorrhage.—Griffiths. (*Practioner*, March, 1877.) Dr. G. was recently consulted in two cases of severe post-partum hemorrhage. In both cases every means had been adopted but unavailingly. It flashed across his mind in the first case to try the effect of ether-spray, and accordingly he directed a large spray across the abdominal walls, along the spine and over the genitals; the uterus at once responded, and the cessation of the hemorrhage was almost immediate. In the second case, he lost no time in adopting a similar treatment, and with an equally successful result. He has consulted several eminent obstetric practitioners in Dublin, and was informed by them that they were not aware that this treatment has been heretofore proposed. The advantages of the ether spray over the application of cold water, and the other means usually adopted to every practitioner of midwifery, must be apparent

D. A. K. S.

Imperforate Hymen with Large Accumulation of Menstrual Fluid.—Winsor.—(*Boston Medical and Surgical Journal*, July 12th, 1877.) On November 4th, 1875, an American girl, unmarried, aged 17, was seized with dysuria, having urinated but once scantily in twenty-four hours. She had no fever, acceleration of pulse, or pain, except during the attempts to urinate. During the previous year had one or two similar attacks, though less severe. She had never menstruated, nor had any periodic symptoms. Had occasional "stomach ache" in the ovarian regions. She was of average height, but thin and rather feeble. Under medical advice she had been kept out of school, and had taken tonics for the past year. The dysuria continued six hours, when she voided a quart of pale inodorous normal urine. Was given antispasmodics. Twelve days later she again suffered from distressing retention of urine. For a week previous micturition had been "troublesome and painful." She was etherized with a view to overcome spasm, if that was the cause, but no urine flowed. On attempting to pass the catheter, the vulvar opening was found filled by a protruding tumor tense, fluctuating and closely resembling the unruptured membranes of the second stage of labor. The margin of the vaginal tumor was continuous with the walls of the vagina. Palpation showed the abdomen occupied by a median tumor extending above the umbilicus. Here, then, was a firm imperforate hymen, and behind it fluid. The finger in the rectum and a hand over the abdominal portion of the tumor, showed it to be continuous and fluctuating. The bladder was emptied with the catheter without diminishing the size of the tumor. A diagnosis of retained menstrual fluid was made, and Dr. C.E. Buckingham called in consultation and confirmed the diagnosis. Gradual evacuation of the fluid was decided on, and under ether about six ounces of thick, dark brown inodorous fluid was withdrawn through the largest canula of an aspirator, when the canula was withdrawn and the fluid allowed to ooze away. Measurement of the abdominal portions of the tumor showed it, to extend one and a half inches above the umbilicus, and about four inches transversely.

The day following the puncture, December 27th, her pulse was 104, temperature 97°. The discharge kept draining away for four days, when the tumor had diminished at least two-thirds in size, when the discharge became offensive, and a solution of bromo chloralum was injected. That evening the temperature was 103°.

On the fifth day she had great abdominal distress,—face was pinched and anxious; pulse, 110; temperature, 101. Nothing could be passed through the puncture. She was etherized, and an incision made at the seat of puncture that would admit the finger into a roomy vagina, then with scissors the hymen was freely opened up antero-posteriorly and laterally. About a quart of tarry stinking fluid escaped. The vagina and womb were washed out freely with a solution of bromochloralum, and an oiled oakum plug left in the vagina. She was given six grains of quinia four times daily. The next day a petechial eruption was noticed over the trunk on the following day; eight days from the first puncture she had a rigor followed by severe pain in the right iliac region, and symptoms indicating blood-poisoning. Under the use of brandy, salicylic acid and nourishing food, in the course of a few weeks she recovered from these symptoms without any abscess having formed. She menstruated scantily, February 3d and March 8th; and normally, April 11th, (fourteen weeks after the operation) and every month since. She now weighs thirty-four pounds more than when she left her bed, and is in every way healthy and happy. It has been suggested as an improvement in the operative treatment of such cases, that after the *first* withdrawal of fluid some disinfecting solutions be injected to avoid the danger of septicæmia, while the advantages of gradual drainage are still retained. The risk attending sudden and complete evacuation, is said to be less from so-called "shock" than from the peritonitis induced by laceration of existing adhesions, as the womb suddenly settles down in the pelvis. D. A. K. S.

PREMATURE DELIVERY PROVOKED BY THE SOFT SOUND.—M. Marchal.—(*Gazette Obstétricale*, May 5, 1877.) M. Marchal has brought on labor successfully, 236 days after the end of the last mentioned period, in a woman 37 years of age, presenting an antero-posterior diameter of 8 centimetres (3-15 inches) at the superior strait, and in whom two former labors had been terminated by cephalotripsy.

The proceeding of M. Marchal consists in the introduction of a gum elastic sound, provided with a mandrel which is withdrawn as the top of the sound enters the uterine orifice. If necessary, an intra-uterine injection may be used to separate the membranes over a greater extent of surface.

M. Granjean, following the example of M. Staltz, has made use of a male metallic sound, for the same purpose, obtaining complete success. L. W. C.

THE PROPHYLACTIC TREATMENT OF PLACENTA PRÆVIA.—Thomas.—(*The American Practitioner*, May, 1877.) Induction of premature delivery after the period of viability of the child, is the only method at present which will enable the obstetrician to avert the evils attendant upon the three last months of utero-gestation. In every case of placenta prævia, where the lives of both mother and child are threatened by repeated hemorrhages, premature delivery should be induced. The only contra-indication claimed to premature delivery is, that less than nine months of intra-uterine life fails to give a child as good a chance for life as one arrived at full term has. Dr. T. says that, "an eight months' child out of the uterus, depending upon pulmonary respiration, has brighter prospect for life than one in the cavity depending for aeration of its blood upon a crippled and bleeding placenta." The author reports eleven cases of premature delivery conducted by him, being the number in full of all his cases of placenta prævia treated by the prophylactic method. Of the eleven cases, labor occurred at the eighth month in seven; of these, one mother died of septicemia, and two children were still-born; of the rest of the cases, (4) two were delivered a little before full term ($8\frac{1}{2}$ m.). One child was born dead, and one was still-born. In all cases of placenta prævia, premature delivery should not be induced, before the period of viability of the child, unless the life of the mother is threatened. Barnes' dilators were employed to dilate the cervix, as a preparatory step to delivery. W. F. L.

